# Identification of human viral protein-derived ligands recognized by individual MHCI-restricted T-cell receptors

**DOI:** 10.1038/icb.2016.12

**Published:** 2016-03-29

**Authors:** Barbara Szomolay, Jie Liu, Paul E Brown, John J Miles, Mathew Clement, Sian Llewellyn-Lacey, Garry Dolton, Julia Ekeruche-Makinde, Anya Lissina, Andrea J Schauenburg, Andrew K Sewell, Scott R Burrows, Mario Roederer, David A Price, Linda Wooldridge, Hugo A van den Berg

**Affiliations:** 1 Warwick Systems Biology Centre, University of Warwick, Coventry, UK; 2 Institute of Infection and Immunity, Cardiff University School of Medicine, Heath Park, Cardiff, UK; 3 Vaccine Research Center, National Institute of Allergy and Infectious Diseases, National Institutes of Health, Bethesda, MD, USA; 4 QIMR Berghofer Medical Research Institute, Brisbane, Queensland, Australia; 5 Wright Fleming Wing, St Mary's Campus, Imperial College, London, UK; 6 Faculty of Health Sciences, University of Bristol, Biomedical Sciences Building, Bristol, UK

## Abstract

Evidence indicates that autoimmunity can be triggered by virus-specific CD8^+^ T cells that crossreact with self-derived peptide epitopes presented on the cell surface by major histocompatibility complex class I (MHCI) molecules. Identification of the associated viral pathogens is challenging because individual T-cell receptors can potentially recognize up to a million different peptides. Here, we generate peptide length-matched combinatorial peptide library (CPL) scan data for a panel of virus-specific CD8^+^ T-cell clones spanning different restriction elements and a range of epitope lengths. CPL scan data drove a protein database search limited to viruses that infect humans. Peptide sequences were ranked in order of likelihood of recognition. For all anti-viral CD8^+^ T-cell clones examined in this study, the index peptide was either the top-ranked sequence or ranked as one of the most likely sequences to be recognized. Thus, we demonstrate that anti-viral CD8^+^ T-cell clones are highly focused on their index peptide sequence and that ‘CPL-driven database searching' can be used to identify the inciting virus-derived epitope for a given CD8^+^ T-cell clone. Moreover, to augment access to CPL-driven database searching, we have created a publicly accessible webtool. Application of these methodologies in the clinical setting may clarify the role of viral pathogens in the etiology of autoimmune diseases.

CD8^+^ T cells recognize antigens in the form of intracellular protein-derived peptide fragments (8–14 amino acids in length) presented on the cell surface by major histocompatibility complex class I (MHCI) molecules. Although this *modus operandi* enables the elimination of cancerous or infected cells, dysregulated CD8^+^ T-cell immunity can have devastating consequences for the host. For example, it has been proposed that CD8^+^ T cells play a major role in the pathogenesis of common autoimmune diseases, such as type 1 diabetes,^1, 2, 3^ multiple sclerosis^4^ and psoriasis,^5^ where pathogen-derived peptide sequences are thought to drive the expansion of self-reactive T cells capable of mediating tissue damage.^6, 7^ This theory is supported by findings that microbial peptides can induce experimental autoimmune disease in mouse models and that human autoantigen-specific T cells can recognize numerous peptides, some of which are microbial in origin.^8, 9^ Moreover, in certain disease states, the presence of monoclonal/oligoclonal CD8^+^ T-cell expansions with a late-differentiation phenotype, sometimes referred to as large granular lymphocytes (LGLs), is suggestive of an exaggerated antigen-specific response.^10^ Such expansions are a characteristic feature of T-LGL leukemia^11, 12, 13^ and can be triggered by certain drugs, notably protein tyrosine kinase inhibitors.^14, 15^ CD8^+^ T-cell expansions are also observed in autoimmune diseases such as rheumatoid arthritis^16^ and aplastic anemia.^17^ It is possible that viral antigens drive these pathogenic CD8^+^ T-cell expansions, which subsequently crossreact with self-derived peptide-MHCI (pMHCI) molecules to precipitate clinical disease.

Although it is clear that CD8^+^ T cells play an important role in health and disease, relatively little is known about the microbial and self-derived ligands involved in these processes. This lack of knowledge can to a large extent be attributed to the complexity of the peptide repertoire recognized by individual T-cell receptors (TCRs). Estimates suggest that there are ~25 million unique TCRs in the human repertoire,^18^ each with the potential to recognize up to 1 million different MHC-bound peptides.^19, 20^ Such promiscuous recognition has been deemed essential for effective immunity, as a relatively limited repertoire of TCRs must provide adequate coverage against a vast array of different pMHC molecules.^21^ Indeed, a given TCR may not only interact productively with ligands similar to the index peptide that triggered the initial response, but also with ligands that are unrelated in sequence,^22^ indicating that effective characterization of the cognate ligand repertoire must take the entire peptide universe into account without bias. A promising approach that satisfies these *desiderata* is combinatorial peptide library (CPL) scanning, which can be combined with biometrical analysis to identify naturally occurring ligands.^23, 24^

Although the set of peptides recognized by an individual TCR can be vast, not all of these sequences will be present in the naturally occurring MHC-presentable peptide repertoire. Novel approaches are therefore required to identify biologically relevant ligands. Ideally, such an approach should incorporate: (i) an assessment of peptide length specificity;^25^ (ii) an unbiased framework applicable to all TCRs irrespective of specificity and MHC restriction; (iii) rapid throughput for clinical applicability; and (iv) an accurate end point for the reliable classification of response-evoking ligands *in vivo*.

Here, we develop and validate a strategy to examine the peptide repertoire recognized by individual MHCI-restricted TCRs. Raw datasets from length-matched CPL scans were used to rank peptides occurring in curated databases of viral pathogen or human self origin. The predictive value of the scoring method was then validated by measuring functional sensitivity for a selection of peptides spanning a range of predicted agonist likelihood values. This approach enabled us to identify the original viral determinant for a CD8^+^ T-cell response. We envisage that ‘CPL-driven database searching' will find clinical utility across a range of immune-mediated diseases with currently unknown antigenic triggers.

## Results

### Development and validation of an effective approach to identify natural ligands recognized by individual MHCI-restricted TCRs

Pathogenic CD8^+^ T-cell expansions may originate as an initially protective response to a viral antigen that results in immune-mediated disease, caused by subsequent crossreactivity with a self-derived pMHCI molecule. The need therefore arises to identify viral ligands that trigger CD8^+^ T-cell expansions of unknown specificity. To develop and validate an approach to this problem, we interrogated a CD8^+^ T-cell clone (E7NLV) specific for the immunodominant HLA A*0201-restricted human cytomegalovirus (HCMV) pp65_495–503_ epitope NLVPMVATV; HCMV is a member of the herpesvirus family that has been implicated in the pathogenesis of common CD8^+^ T-cell-mediated diseases.^26^

**Figure 1:**
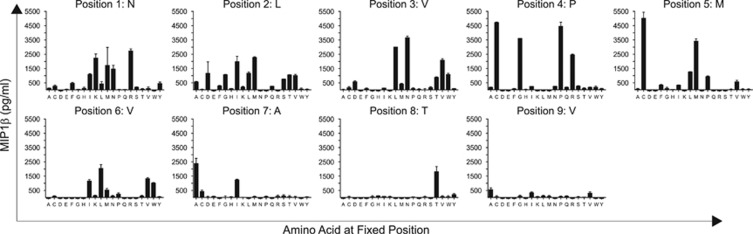
Nonamer CPL scan of E7NLV. 6 × 10^4^ C1R-A*0201 target cells were pulsed in duplicate with mixtures from a 9-mer CPL scan (100 μM) at 37 °C. After 2 h, 3 × 10^4^ E7NLV CD8^+^ T cells were added and incubated overnight. The supernatant was then harvested and assayed for MIP1β by ELISA.

MHCI-restricted TCRs display an explicit preference for peptide length, as attested by the fact that screening with CPLs of non-preferred length elicits a minute number of positive responses, whereas screening with CPLs of the preferred length elicits responses at every peptide position, allowing the molecular recognition landscape for each individual TCR to be mapped in detail.^25^ Accordingly, we scanned E7NLV with a nonamer CPL to determine the amino acid preferences across the peptide backbone. Multiple responses were observed at the majority of positions, indicative of a crossreactive TCR with a propensity for degenerate peptide recognition (Figure 1). Although CPL scan data can be used to perform BLAST or motif-based searches directly, these approaches generate peptide lists without quantifying the likelihood of recognition. We took an alternative approach that uses raw peptide length-matched CPL scan data to search large protein databases, producing lists of potential peptide agonists ranked in order of likelihood of recognition. This ranking was achieved by assigning an agonist likelihood score Λ, as defined below by equation (8), to each subsequence in a protein database comprising the majority of known viruses regardless of host tropism.

**Figure 2:**
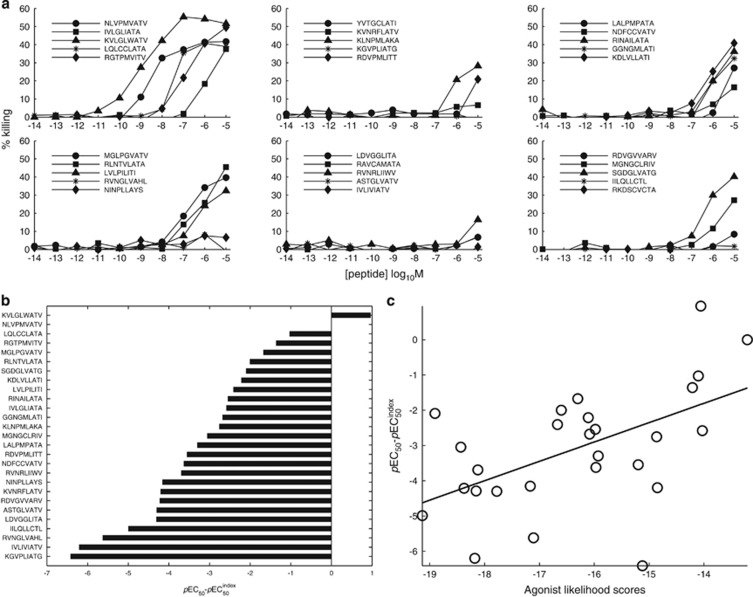
Recognition of 30 randomly chosen and uniformly distributed peptides by the E7NLV clone. (**a**) 1 × 10^3^ C1R-A*0201 target cells per condition were pulsed with a panel of 30 peptides over a range of concentrations in duplicate for 1 h at 37 °C. 2 × 10^3^ E7NLV CD8^+^ T cells were then added to achieve an E/T ratio of 2:1. Cytotoxic activity was measured via chromium release from C1R-A*0201 target cells as described in the Methods. (**b**) Relative functional sensitivity (Δ*p*EC_50_) for the same 30 peptides compared with index (Δ*p*EC_50_=0). (**c**) Scatter plot of Δ*p*EC_50_ versus Λ for the same 30 peptides.

To validate the accuracy of this approach, 30 peptide sequences were chosen by uniform sampling without repetition such that their Λ-values spanned ~6 orders of magnitude. Sampling was implemented using the MATLAB datasample command; when there were five different peptides in each bin for a given order of magnitude, the sampling algorithm halted. The peptides were synthesized and a functional sensitivity assay was performed. Figure 2a shows the functional response of E7NLV to the 30 selected peptides. A broad range of recognition was observed, with 15 out of 30 peptides acting as good agonists and 6 out of 30 peptides acting as strong agonists. The functional sensitivity of the clone for each peptide was expressed as *pEC*_50_, the common cologarithm of the 50% efficacy concentration. Relative functional sensitivity (Δ*p*EC_50_) was calculated by subtracting the *p*EC_50_ of the index peptide from the *p*EC_50_ of the given peptide (Figure 2b). We examined the statistical dependence between Λ and Δ*p*EC_50_ for all 30 randomly chosen peptides using Spearman's rank correlation test. The correlation for the peptides shown in Figure 2c was 0.49, which is significant at the 2% level, and the corresponding linear regression of Δ*p*EC_50_ on Λ was significant at the 1% level. These results demonstrate that CPL-driven database searching can accurately identify peptide sequences recognized by individual TCRs.

### CPL-driven database searching can identify viral ligands that initiate CD8^+^ T-cell responses

**Figure 3:**
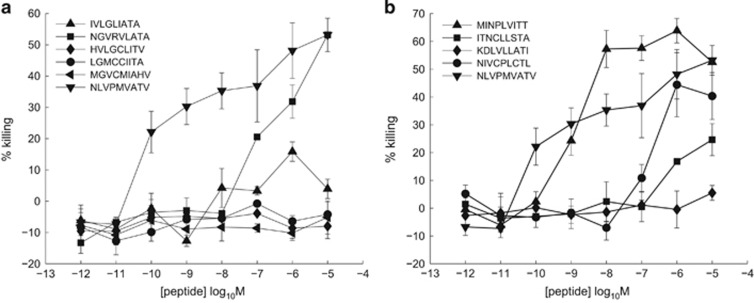
E7NLV recognition of the top 6 peptides (**a**) and peptides ranked 7–10 (**b**) from the human pathogen database. In **b**, peptide recognition is compared with index (black upside–down triangles). Cytotoxic activity was measured via chromium release from C1R-A*0201 target cells as described in the Methods.

We next assessed whether our approach could be used to identify the original viral ligand that drove the initial expansion of a given clonotype, independently of prior knowledge of this ligand or the identity of the infectious agent. For this purpose, we constructed a database containing all human viral pathogens, as well as all zoonotic viruses capable of or suspected to be capable of infecting humans, to the best of our knowledge and judgement. Each of the 1 872 417 unique nonamer peptide sequences in this database of 10 733 distinct viral proteins was assigned a Λ-value according to equation (8), with parameter values derived from a nonameric CPL scan of clone E7NLV. These sequences were ranked by Λ, which represents the likelihood of recognition by this clone. The index peptide for E7NLV, NLVPMVATV, was found to be the top-ranked sequence, suggesting that CPL-driven database searching alone can suffice to identify the infectious agent (Figure 3 and Supplementary Table S1A). We then examined the functional response to a further nine top-ranking peptides. In addition to the index, which had been recovered without prior knowledge, five out of the further nine predicted peptides were capable of activating E7NLV, with three being strong agonists (Figure 3). Thus, CPL-driven database searching could select from a comprehensive database not only the pathogen-derived sequence that had driven the initial expansion of clone E7NLV, but also a variety of crossreactive peptide ligands.

### CPL-driven database searching can identify viral ligands recognized by EBV-specific CD8^+^ T cells

To extend the foregoing findings to other specificities, we examined a panel of Epstein–Barr virus (EBV)-specific CD8^+^ T-cell clones. EBV has been implicated in the pathogenesis of autoimmune diseases such as multiple sclerosis.^27^ Our knowledge regarding the etiology of these diseases would therefore be advanced by a method that can define antigen specificity within the associated CD8^+^ T-cell clonal expansions.^28^ We initially focused on clones SB16 and SB12, which are both specific for the immunodominant HLA A*0201-restricted BMFL1_280–288_ epitope GLCTLVAML (Table 1).

**Figure 4:**
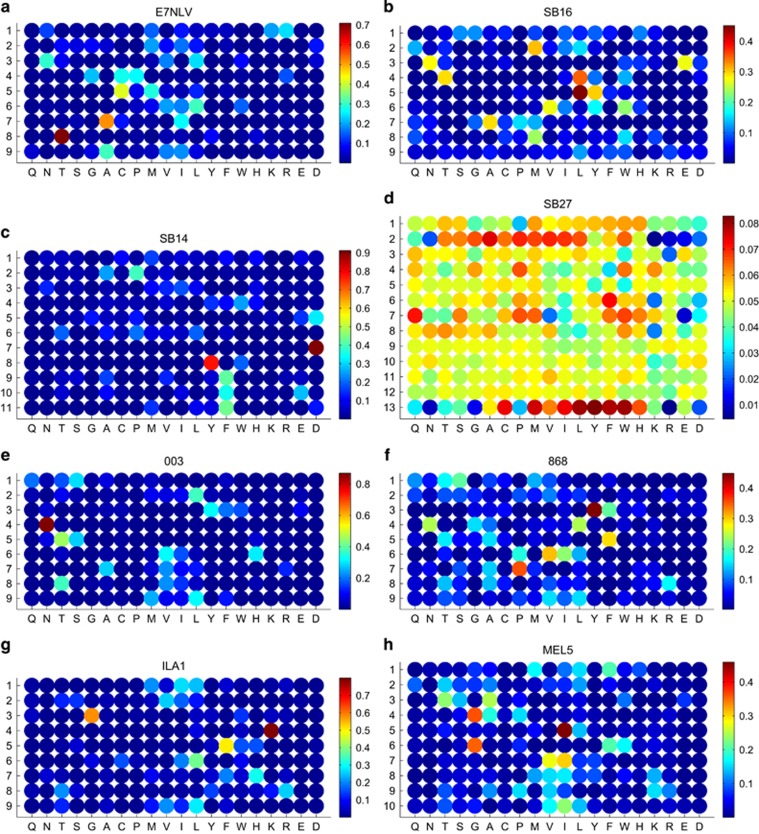
Heat maps summarizing CPL scan data for: E7NLV (**a**), SB16 (**b**), SB14 (**c**), SB27 (**d**), 003 **(e**), 868 (**f**), ILA1 (**g**) and MEL5 (**h**). CPL scan data are normalized in each row so that the values range from high (red) to low (blue); the maximum intensity is the largest of all red values in the rows. Amino acids are grouped according to their physicochemical properties as follows: polar, uncharged amines: Q, N; polar, uncharged alcohols: T, S; small: G, A, C; hydrophobic: A–H; aliphatic: V, I, L; aromatic: Y, F, W, H; large: F, W; charged basic: H, K, R; and charged acidic: E, D.

A nonamer CPL scan of the SB16 clone revealed multiple hits across the peptide backbone with dominant responses at each position (Figure 4 and Supplementary Figure S1). The raw CPL scan dataset was used to conduct a CPL-driven search of the human viral database. Supplementary Table S1B lists the nonameric peptide sequences ranked as the 20 sequences most likely to be recognized out of a database of 1 872 417 different nonameric sequences. The index peptide sequence for this clone was found to have the largest Λ-value, consistent with the result obtained for E7NLV (Supplementary Table S1A). We next performed a nonamer CPL scan with clone SB12 (Table 1). In contrast to the result obtained with SB16, we did not detect responses that rose substantially above background, except at position 8, where a response to methionine was observed (Supplementary Figure S2); these results were too scanty to permit a meaningful CPL-driven database search and suggest that SB12 is highly ligand-specific, recognizing only a small number of peptides. This characteristic may be shared with other anti-viral CD8^+^ T cells, as we observed a similar result with ALF3, a CD8^+^ T-cell clone specific for the immunodominant HLA A*0201-restricted influenza A M1_58–66_ epitope GILGFVFTL (Table 1 and Supplementary Figure S3).

CD8^+^ T cells specific for longer epitopes play a major role in the response against EBV.^29^ We therefore examined two well-characterized clones with different EBV-derived peptide length preferences. Clone SB14 is specific for the immunodominant HLA B*3508-restricted EBNA1_407–417_ epitope HPVGEADYFEY (Table 1). Accordingly, we performed an 11-mer CPL scan on this clone (Figure 4 and Supplementary Figure S4). A CPL-driven search of the human viral database predicted that the 2 most likely recognized 11-mer peptide sequences were HPVAEADYFEY and HPVGDADYFEY (4A and 5D variants of the index peptide), with the index peptide itself ranking third in the list (Supplementary Table S1C). Clone SB27 is specific for the HLA B*3508-restricted BZFL1_52–64_ epitope LPEPLPQGQLTAY and exhibits a strong preference for 13-mer peptides.^25^ A 13-mer CPL scan of this clone revealed a high degree of crossreactivity (Ekeruche-Makinde *et al.*^25^ and Figure 4), but notwithstanding this apparent promiscuity, the index peptide still ranked twelfth in a CPL-driven search of the human viral database (Supplementary Table S1D).

Collectively, these findings demonstrate that viral peptide specificities can be identified efficiently by means of peptide length-matched CPL-driven database searching. In general, anti-viral CD8^+^ T cells appear to be highly focused on their index peptide sequence, albeit with an inherent degree of crossreactivity.

### CPL-driven database searching can identify variants recognized by HIV-1-specific CD8^+^ T cells

To examine the utility of CPL-driven database searching in other viral infections, we studied two previously described TCRs specific for the immunodominant human immunodeficiency virus type 1 (HIV-1)-derived HLA A*0201-restricted p17 Gag_77–85_ epitope SLYNTVATL (Table 1). Consistent with the results above, a CPL-driven search of the human viral database using nonamer CPL scan data from the 003 clone identified the index sequence as the most likely agonist (Figure 4 and Supplementary Figure S5; Supplementary Table S1E). Moreover, a number of epitope variants were predicted, many of which have been verified at the functional level.^30, 31^ Similar results were obtained with the 868 TCR, in this case scanning primary CD8^+^ T cells transduced with the corresponding lentiviral construct (Figure 4 and Supplementary Figure S6; Supplementary Table S1F). CPL-driven database searching can therefore predict epitope variant crossreactivity patterns, which may prove useful in the assessment of CD8^+^ T-cell responses against highly variable viruses.

### Extending the approach to the identification of self ligands targeted by CD8^+^ T cells

In addition to the identification of virus-derived epitopes, it would be advantageous if CPL-driven database searching could reveal self-derived peptide targets, as this would provide a means to identify the antigenic proteins involved in autoimmune diseases and, moreover, to discover novel cancer epitopes recognized by CD8^+^ T cells. To this end, we created a human protein database as described below. The CD8^+^ T-cell clones ILA1 and MEL5 were chosen because they are both specific for epitopes derived from self proteins. In particular, ILA1 is specific for the HLA A*0201-restricted human telomerase reverse transcriptase (hTERT)_540–548_ sequence ILAKFLHWL (Table 1). A nonamer CPL scan previously carried out for ILA1^25, 32^ was used to conduct a CPL-driven search of the human protein database (Figure 4). The index peptide was ranked by Λ as the eighth most likely nonameric peptide sequence to be recognized by the ILA1 TCR, suggesting that this approach is capable of identifying self-derived ligands targeted by TCRs (Supplementary Table S2A).

However, a previous study indicated that ILAKFLHWL is not expressed at the cell surface,^33^ and therefore ILA1 may not be typical of a self-reactive TCR that has survived negative selection. To address this potential confounder, we studied MEL5, which recognizes the HLA A*0201-restricted Melan-A epitope EAAGIGILTV.^34^ A nonamer CPL scan, which had previously been carried out for MEL5,^25, 34^ was used to conduct a CPL-driven search of the human protein database (Figure 4). The EAAGIGILTV sequence did not appear in the top 20 peptides predicted to activate MEL5 (EAAGIGILTV ranked fifty-fifth; Supplementary Table S2B). It is possible that self-reactive TCRs are not focused on their index peptide to the same degree as virus-specific TCRs and, consequently, the CPL-driven database searching approach may fail to identify disease-relevant ligands targeted by self-reactive TCRs. The use of smaller disease-specific or organ-specific databases might improve the applicability of our strategy in this context.

### Overview of CPL-driven database screening and development of a webtool

**Figure 5:**
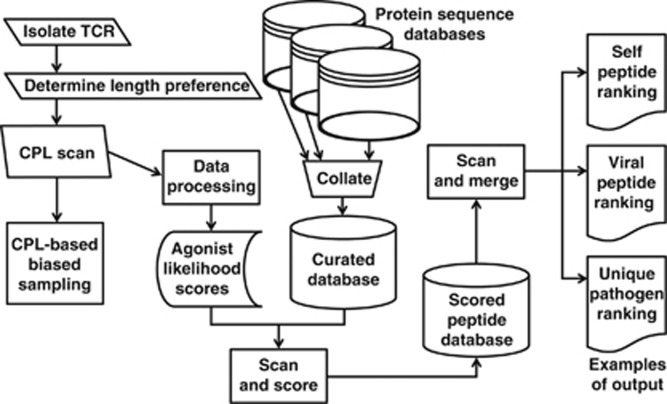
Three-stage strategy to dissect the peptide recognition signature of individual TCRs. To augment community-wide access to CPL-driven database searching, we have created a dedicated webtool as part of the WSBC webtools framework.

Every TCR is characterized by a unique peptide recognition signature, which comprises three different components: (i) a peptide length preference; (ii) the number of peptides recognized at this preferred length; and (iii) the sequence identity of these peptide agonists, which may be either pathogen-derived or self-derived.^20, 25^ Figure 5 provides an overview of a three-stage strategy to dissect the peptide recognition signature of a given TCR. In the first stage, peptide length preference is determined by examining functional recognition of a ‘sizing scan' comprising random peptide libraries of different lengths (*x*_8_, *x*_9_, *x*_10_, *x*_11_, *x*_12_, *x*_13_, where *x* denotes any of the 19 L-amino acids excluding cysteine).^25^ In the second stage, CPL-biased sampling^19^ is used to quantify the number of recognized peptides. In the third stage, which is the novel step introduced in the present study, CPL-driven database searching is used to identify antigen specificity. To augment community-wide access to CPL-driven database searching, we created a dedicated webtool as part of the WSBC webtools framework.

## Discussion

Although CD8^+^ T cells protect the human body from countless intracellular pathogens and malignant processes, they are also heavily implicated in the etiology of life-threatening and incurable immune-mediated diseases. It is conceivable that non-self antigens elicit CD8^+^ T-cell responses that are initially target-appropriate but subsequently become pathogenic as a consequence of crossreactivity with self epitopes. In this study, we developed and validated a technique termed ‘CPL-driven database searching', which allows for the reliable identification of cognate viral epitopes recognized by CD8^+^ T cells with as yet unknown specificities. Initially, we constructed a viral database containing human and zoonotic viruses (with an established or potential ability to infect humans). This database was curated to include non-redundant and reviewed sequences only and contained a total of 10 733 viral proteins. We then selected a panel of herpesvirus-specific CD8^+^ T-cell clones spanning two different restriction elements (HLA A*0201 and HLA B*3508) and a range of epitope lengths (9–13 amino acids). As CD8^+^ T cells typically display an explicit preference for peptide length,^25^ we focused our attention on peptide length-matched CPL scans to determine the amino acid preferences of each clone at each peptide position. The majority of clones in our panel elicited strong responses with distinct preference hierarchies at each position of the peptide. CPL scan datasets were used to assign an agonist likelihood score (Λ) to each peptide of preferred length in the entire human viral database, thereby producing a list of potential peptide ligands in order of likelihood of recognition. For all herpesvirus-specific clones tested, the index epitope ranked in the top 20 most likely recognized peptide sequences from the entire human viral database, even for the highly crossreactive CD8^+^ T-cell clone SB27, which is specific for an EBV-derived HLA B*3508-restricted 13-mer epitope. These findings indicate that anti-viral CD8^+^ T cells display high levels of functional sensitivity for the index epitope, consistent with an efficient *in vivo* selection process based on interclonal competition for antigen.^35^

It is noteworthy that one EBV-specific CD8^+^ T-cell clone failed to respond to any CPL scan mixtures except M@8 and was therefore not suitable for further analysis. This difficulty could be emblematic of a small subset of CD8^+^ T-cell clones that are highly focused on the salient ligand and therefore fail to respond to a sufficiently large number of peptides within the CPL mixtures. CPL-driven database searching is not applicable to such TCRs, which behave as ‘non-responders' in the context of a CPL scan. Moreover, to ensure the accuracy of CPL-driven database searching, experimental CD8^+^ T-cell populations must be monoclonal and must be subjected to multiple replicates of each scan.

The viral pathogen and human proteome databases were collated and curated with a view to making them as exhaustive as possible. It may be considered that the relatively small size of the former database contributes to the success of our method, but this is not the case. Indeed, when the two databases were merged, comparable rankings were obtained. In terms of relative rank, the merged database was slightly better for most TCRs, indicating that the ranks derived from the viral database are actually more conservative. Notwithstanding these advantages, the identification of relevant epitopes in the human proteome database is much more challenging, likely due to the fact that even physiologically relevant autoimmune epitopes are typically recognized at low levels of functional sensitivity. Work is in progress to build and test similar databases for bacterial and fungal pathogens. These will be added to the webtool on completion.

It is known that HIV-1 can escape from the CD8^+^ T-cell response via single point mutations in key epitopes.^36^ If CD8^+^ T cells are inherently crossreactive, why does the HIV-1-specific CD8^+^ T-cell response exhibit such exquisite specificity? Our findings point to a possible explanation. The HIV-1-specific TCRs examined in this study (003 and 868) were strongly focused on their index peptide sequence, increasing the probability that epitope mutation will result in loss of recognition. In contrast, CD8^+^ T cells with a more crossreactive phenotype are associated with delayed disease progression.^37, 38^ It is therefore feasible that CPL-driven database searching will provide a useful tool to delineate the requirements for effective CD8^+^ T-cell-mediated immunity against HIV-1.

CPL-driven database searching may also assist in the identification of self-derived epitopes, such as those targeted by autoimmune or cancer-specific CD8^+^ T cells. Indeed, similar approaches to ligand hunting in these settings have been described previously.^39, 40^ Although we successfully identified the index peptide for the ILA1 clone, it has been shown that the cognate epitope is not expressed on the surface of HLA A*0201^+^ hTERT^+^ cancer cell lines,^33^ which renders it less likely that this epitope is expressed in the thymus. If so, ILA1 will not have been subjected to negative selection in the thymus, which could account for the highly focused phenotype of this clone. In contrast, the index EAAGIGILTV peptide recognized by the MEL5 clone was ranked at position 55. It may therefore be more challenging to identify *bona fide* self-derived epitopes that are expressed in the thymus. Generation of disease-specific or organ-specific protein databases might circumvent this problem by narrowing the search for relevant epitopes, but further work is required to test such focused screening strategies.

In summary, we have developed and validated an approach that can be used to dissect the peptide recognition signature of any given TCR. Accordingly, we anticipate that CPL-driven database searching will find clinical utility across a range of diseases.

## Methods

### Cells

Ten human CD8^+^ T-cell clones spanning eight different specificities were used in this study (Table 1). The index peptides for the following CD8^+^ T-cell clones are derived from viral proteins: E7NLV, SB16, SB12, ALF3,^41^ SB14,^42^ SB27,^43^ 003^44^ and 868.^44^ The index peptides for the remaining two CD8^+^ T-cell clones, ILA1^45^ and MEL5,^46^ are derived from human self proteins. Clone E7NLV is specific for the HLA A*0201-restricted HCMV-derived pp65 epitope NLVPMVATV (residues 495–503). Four clones are specific for epitopes derived from EBV proteins: SB16 and SB12, which both recognize the HLA A*0201-restricted BMLF1 epitope GLCTLVAML (residues 280–288); SB14, which recognizes the HLA B*3508-restricted EBNA1 epitope HPVGEADYFEY (residues 407–417); and SB27, which recognizes the HLA B*3508-restricted BZLF1 epitope LPEPLPQGQLTAY (residues 52–64). Clone ALF3 is specific for the HLA A*0201-restricted influenza A-derived M1 epitope GILGFVFTL (residues 58–66; Clement *et al.*^41^). The 003 and 868 TCRs are both specific for the HLA A*0201-restricted HIV-1-derived p17 Gag epitope SLYNTVATL (residues 77–85). Clone ILA1 is specific for the HLA A*0201-restricted hTERT-derived sequence ILAKFLHWL (residues 540–548), and clone MEL5 is specific for the Melan-A-derived heteroclitic sequence ELAGIGILTV (residues 26–35). All CD8^+^ T cells were maintained in RPMI 1640 containing 100 U ml^−1^ penicillin, 100 mg ml^−1^ streptomycin, 2 mM L-glutamine and 10% heat-inactivated fetal calf serum (all Life Technologies, Paisley, UK), supplemented with 2.5% Cellkines (Helvetica Healthcare, Geneva, Switzerland), 200 IU ml^−1^ interleukin (IL)-2 and 25 ng ml^−1^ IL-15 (both PeproTech, Rocky Hill, CT, USA). C1R-A*0201 and T2-B*3508 target cells were generated and maintained as described previously.^47^

### CPL scans

CPL libraries were synthesized in a positional scanning format (Pepscan Presto Ltd, Lelystad, Netherlands). All CPL scans were performed as described previously.^19, 25, 32^ Briefly, 6 × 10^4^ target cells were incubated with various library mixtures (at 100 μM) in duplicate for 2 h at 37 °C. After peptide pulsing, 3 × 10^4^ clonal CD8^+^ T cells were added and the plates were incubated overnight to 37 °C. Supernatants were then collected and assayed for MIP1β content by enzyme-linked immunosorbent assay (ELISA; R&D Systems, Abingdon, UK).

### Functional sensitivity assays

For MIP1β ELISA assays, 6 × 10^4^ target cells were pulsed with peptide at the indicated concentrations for 2 h at 37 °C. Subsequently, 3 × 10^4^ clonal CD8^+^ T cells were added and the plates were incubated overnight at 37 °C. Supernatants were then collected and assayed for MIP1β by ELISA. For lysis assays, target cells were loaded with ^51^Cr for 1 h and then plated at 5000 cells per well in 75 μl medium. Clonal CD8^+^ T cells were added to a total volume of 150 μl. Target cells alone were used to measure spontaneous release and Triton X-100 was used to measure total release. After incubation for 4 h at 37 °C, 15 μl supernatant per well was harvested and mixed with 150 μl OptiPhaseSupermix Scintillation Cocktail (Perkin-Elmer, Waltham, MA, USA). Release of ^51^Cr was measured with a Microbeta Counter (Perkin-Elmer) and the resulting data were used to calculate % specific lysis according to the formula:


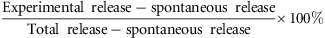


Functional sensitivity was determined as described previously;^19^ it is expressed as *p*EC_50_, the cologarithm to base 10 of the 50% efficacy concentration as determined from dose–response experiments in which antigen-presenting cells were incubated with agonist across a series of dilutions.

### Derivation of the agonist likelihood score method

The functional sensitivity of a clone of interest to a given peptide ligand depends on several factors, a key parameter being the rate at which a single pMHCI copy elicits TCR triggering.^48, 49^ We quantify TCR degeneracy as the probability that the functional sensitivity of a given TCR to a randomly chosen ligand exceeds a given value *ω*;^50^ this probability expresses the degeneracy at *ω*. Let *w*_*ij*_ denote the functional sensitivity of TCR *i* to peptide *j*, where 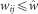 for all *i* and *j* (with 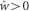). Besides being a function of TCR *i* and pMHCI species *j*, functional sensitivity depends on additional factors such as cellular differentiation and coreceptor expression; we assume such additional parameters to be uniform across the experiments performed in the present study. If *n* is the length of the peptides considered, then 20^*n*^ is the size of the peptide universe (the total number of distinct peptides). Given a peptide subset 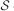 of the peptide universe, the general question of epitope prediction can be framed as follows: for a clone of interest *i*, determine the quantity 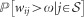, that is, the probability that *w*_*ij*_ exceeds a set value 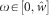, when it is known that the peptide ligand *j* belongs to this set 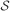. For a peptide *j* selected at random, we have 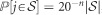, where 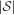 denotes the cardinality of 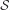. Then, by Bayes' Rule,


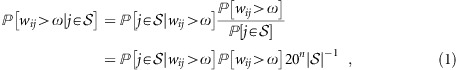


which expresses the problem in the empirically more accessible probability 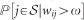, that is, the chance that a randomly selected peptide *j* belongs to 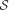, given that the functional sensitivity *w*_*ij*_ to this peptide is at least *ω*. A direct estimate of this probability could be obtained by subjecting randomly generated peptides to a functional sensitivity assay and verifying whether they also belong to 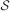. However, this would be highly inefficient and would require millions of random peptides to be reasonably accurate, as the vast majority would be practically ‘null' (that is, having *p*EC_50_ well below the detection limit). More progress can be made provided that 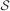 is a cylinder, which is a special type of set, defined as follows: let 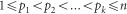 represent a choice of positions in the peptide and let 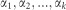 be a choice of amino acid residues for these positions (several or all of these *α*s could be the same amino acid). Then a peptide set of the form


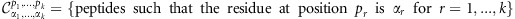


is called a cylinder of rank *k*. For example, the rank-2 cylinder 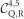 contains all and only the peptides with glutamine at position 4 and arginine at position 5. The cardinality of a rank-*k* cylinder is 20^*n−k*^; in particular, the rank-0 cylinder is the entire peptide universe, as no amino acids are specified at any position, whereas a rank-*n* cylinder contains just one peptide, all positions having been specified.

Given a rank-*k* cylinder 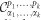, let 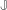 denote a subset of 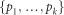, and let 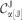 be the lower-order cylinder which has amino acids specified only at the positions in 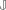, agreeing with the ‘parent' 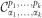 at those positions, that is, 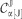 is formed by dropping from 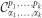 the positions not present in 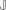. Let 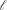 be an integer 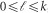, and let 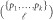 denote the set of all subsets 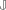 of size 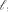, so that cylinders can be decomposed as follows:


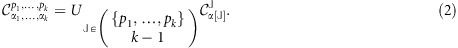


Next, let correlation functions be defined recursively, as follows:


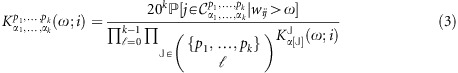


with lowest-order boundary condition 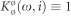 for all 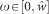. The correlation functions permit an *exact* solution of the epitope prediction problem, since equation (3) implies that


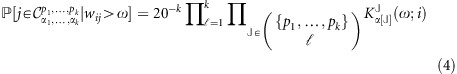


and with equation (1) this can be transformed into the probability that a peptide is an agonist with functional sensitivity of at least *ω*, given that the peptide belongs to some cylinder of interest, which might be of appropriate cardinality, be this almost the entire peptide universe or a singleton set (that is, a rank-*n* cylinder containing only that peptide).

The crucial step is to establish a connection to CPL data. Consider the CPL mixture K@3, in which lysine is fixed at position 3 with all other positions random. This mixture corresponds to the rank-1 cylinder 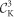. Similar correspondences obtain for all 180 CPL mixtures (in the case *n*=9). Suppose that a peptide contributes appreciably to the CPL read-out whenever *w*_*ij*_ exceeds a certain cut-off value 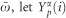 denote the read-out signal obtained when exposing clone *i* to the mixture *α*@*p*, and let 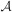 denote the set of 20 amino acids. We then propose the following identification:


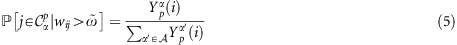


where *p* is the single specified position (with 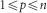) and *α* is the specified amino acid residue at this position. The rank-1 specialization of equation (3) is as follows:


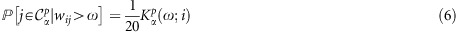


and thus, with *ω* set to 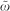, equation (5) provides a way to estimate the lowest-order correlation function from the CPL scan data. The proposed identification, equation (5), rests on somewhat idealized assumptions, since peptides with *w*_*ij*_ values below 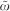 may still contribute if they are presented at high copy numbers on the antigen-presenting cell and, conversely, peptides with high *w*_*ij*_ values may fail to contribute substantially.

In principle, higher-rank correlation functions can be estimated from higher-rank CPLs, which contain library mixtures corresponding to higher-rank cylinders (for example, the mixture K@3&A@5 corresponds to the rank-2 cylinder 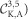), but the number of peptide mixtures required, and hence the cost of the experiment, quickly becomes prohibitive. Rank-1 correlations can nevertheless be informative as long as higher-order correlations do not dominate. Mathematically speaking, the higher-order correlations are neglected by setting the corresponding *K*-values equal to 1; this amounts to a truncation of the exact expansion in equation (4). Combining equations (1) and (5) with the truncated version of equation (4), we obtain the following for a specified peptide 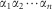:


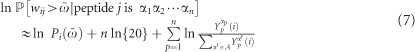


where 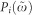 is the unconditional probability that the functional sensitivity of clone *i* for a peptide selected at random exceeds 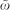. For the purpose of ranking peptides, we can proceed without knowing the value of 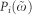, since it merely represents a fixed offset term, once a clone has been fixed. We therefore retain only the last term in equation (7), which we call the agonist likelihood score:


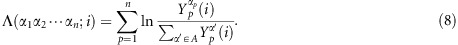


MATLAB scripts were written to scan the human and viral pathogen databases and to evaluate Λ for all potential peptides of the clone-appropriate length in the proteomes (that is, all subsequences of the specified length irrespective of antigen processing constraints). Excluded were those containing one or more unspecified residues, appearing as X in the databases; such cases were rare. NetChop predictions of MHCI binding were also obtained via the publicly available online tool, but these data are not reported here because we did not find that they winnowed the candidate peptide list.

### Data access provisions

#### Databases

Human and viral databases were compiled on the basis of publicly available protein sequence databases provided by NCBI (National Center for Biotechnology Information), UniProt (Universal Protein Resource) and PDB (Protein Data Bank).

The human proteome database was assembled using protein sequence information taken from the following data sources:

(i) File protein.fa.gz from

ftp://ftp.ncbi.nih.gov/genomes/Homo_sapiens/protein/;

(ii) File human.protein.faa.gz from

ftp://ftp.ncbi.nih.gov/refseq/H_sapiens/mRNA_Prot/;

(iii) http://www.uniprot.org/uniprot/ for canonical and isoform sequence data in FASTA format (Protein Knowledgebase search terms: organism:homo sapiens and reviewed:yes);

(iv) http://www.pdb.org/pdb/home/home.do for human protein sequences (search terms: taxonomy:homo sapiens, polymer type:protein, custom tabular report:sequence, macromolecular name, source).

The viral protein database was assembled using protein sequence information taken from the following data sources:

(i) file viral.1.protein.faa.gz from

ftp://ftp.ncbi.nih.gov/refseq/release/viral/;

(ii) file all.faa.tar.gz from

ftp://ftp.ncbi.nih.gov/genomes/Viruses;

(iii) http://www.uniprot.org/uniprot/ for canonical and isoform sequence data in FASTA format (Protein Knowledgebase search terms: host:human and reviewed:yes);

(iv) http://www.pdb.org/pdb/home/home.do for viral protein sequences (search terms: taxonomy:viruses, polymer type:protein, custom tabular report:sequence, macromolecular name, source).

(Remote file names and locations may be subject to change).

The assembled databases were curated to contain non-redundant and certified sequences, comprising 54 886 human and 187 840 viral proteins, respectively. As the viral database was compiled from a variety of data sources, care was taken to ensure that each pathogen was identified by a unique key and that different keys for the same pathogen used in different sources were slaved to the master key. A list of ~250 viral species with known or potential ability to infect humans was compiled and used to restrict the viral database to pathogens that infect humans; avoidance of ambiguity poses a major challenge in the face of issues surrounding the distinction of closely related species of viral pathogens and the frequent use of alternative designations. Ultimately, there were 10 733 non-redundant protein sequences in the database of proteomes of viral pathogens that pose a potential danger to human beings.

#### Webtool

A novel webtool, PI CPL, was written in MATLAB and compiled using the MATLAB Compiler. The binary code was integrated into the WSBC webtools framework, accessible at http://wsbc.warwick.ac.uk/wsbcToolsWebpage. The framework provides a browser-based user interface, from which jobs are launched and run on a multicore computational cluster. Feedback on progress is provided via a webpage if required. Results are presented as a webpage and can also be downloaded for offline viewing. In the case of PI CPL, these consist of a text file containing a list of the top-scoring peptides, which is designed to be viewed as a spreadsheet, plus a heat map image. A database of results is maintained as a job history for logged-on users. To request an account, please e-mail the corresponding author.

## Figures and Tables

**Table 1:** CD8^+^ T-cell clones used in this study

*Clone ID*	*Specificity*	*HLA restriction*	*Minimal epitope*	*Peptide length*	*Reference*
E7NLV	HCMV	A*0201	NLVPMVATV	9	a
SB16	EBV	A*0201	GLCTLVAML	9	a
SB12	EBV	A*0201	GLCTLVAML	9	a
ALF3	Influenza A	A*0201	GILGFVFTL	9	Clement *et al.*^41^
SB14	EBV	B*3508	HPVGEADYFEY	11	Miles *et al.*^42^
SB27	EBV	B*3508	LPEPLPQGQLTAY	13	Tynan *et al.*^43^
003	HIV-1	A*0201	SLYNTVATL	9	Goulder *et al.*^44^
868	HIV-1	A*0201	SLYNTVATL	9	Goulder *et al.*^44^
ILA1	Telomerase	A*0201	ILAKFLHWL	9	Laugel *et al.*^45^
MEL5	Melan-A	A*0201	ELAGIGILTV	10	Cole *et al.*^46^

Abbreviations: EBV, Epstein–Barr virus; HCMV, human cytomegalovirus; HIV, human immunodeficiency virus; TCR, T-cell receptor.

a First description of this CD8^+^ T-cell clone. The parental clone was used in all cases except for 868, where primary CD8^+^ T cells were transduced to express the 868 TCR.
