# Supplementary material for: Identification of human viral protein-derived ligands recognized by individual MHCI-restricted T-cell receptors
Source: Immunol Cell Biol. 2016 Mar 29;94(6):573–82. doi: 10.1038/icb.2016.12 (PMC4943067; doi:10.1038/icb.2016.12)
Supplement: Supplementary Information [file icb201612x1.pdf]

Supplemental File

Identification of human viral protein-derived ligands recognized by individual major histocompatibility complex class I (MHCI)-restricted T-cell receptors

Barbara Szomolay, Jie Liu, Paul E. Brown, John J. Miles, Mathew Clement, Sian Llewellyn-Lacey, Garry Dolton, Julia Ekeruche-Makinde, Anya Lissina, Andrea J. Schauenburg, Andrew K. Sewell, Scott R. Burrows, Mario Roederer, David A. Price, Linda Wooldridge*, Hugo A. van den Berg*

Corresponding author: L. Wooldridge
Email: linda.wooldridge@bristol.ac.uk
*LW and HAB contributed equally to this manuscript.

SUPPLEMENTAL FIGURE LEGENDS

Figure S1: CPL scan of SB16 CD8$^+$ T-cells: 6x10^4 target cells expressing HLA A*0201 were pulsed in duplicate with mixtures from a 9-mer CPL scan (100 µM) at 37ºC. After 2 hours, 3x10^4 SB16 CD8$^+$ T-cells were added and incubated overnight. The supernatant was then harvested and assayed for MIP1β by ELISA.

Figure S2: CPL scan of SB12 CD8$^+$ T-cells: 6x10^4 target cells expressing HLA A*0201 were pulsed in duplicate with mixtures from a 9-mer CPL scan (100 µM) at 37ºC. After 2 hours, 3x10^4 SB12 CD8$^+$ T-cells were added and incubated overnight. The supernatant was then harvested and assayed for MIP1β by ELISA.

Figure S3: CPL scan of ALF3 CD8$^+$ T-cells: 6x10^4 target cells expressing HLA A*0201 were pulsed in duplicate with mixtures from a 9-mer CPL scan (100 µM) at 37ºC. After 2 hours, 3x10^4 ALF3 CD8$^+$ T-cells were added and incubated overnight. The supernatant was then harvested and assayed for MIP1β by ELISA.

Figure S4: CPL scan of SB14 CD8$^+$ T-cells: 6x10^4 target cells expressing HLA B*3508 were pulsed in duplicate with mixtures from an 11-mer CPL scan (100 µM) at 37ºC. After 2 hours, 3x10^4 SB14 CD8$^+$ T-cells were added and incubated overnight. The supernatant was then harvested and assayed for MIP1β by ELISA.

Figure S5: CPL scan of 003 CD8$^+$ T-cells: 6x10^4 target cells expressing HLA A*0201 were pulsed in duplicate with mixtures from a 9-mer CPL scan (100 µM) at 37ºC. After 2 hours, 3x10^4 003 CD8$^+$ T-cells were added and incubated overnight. The supernatant was then harvested and assayed for MIP1β by ELISA.

Figure S6: CPL scan of 868 CD8$^+$ T-cells: 6x10^4 target cells expressing HLA A*0201 were pulsed in duplicate with mixtures from a 9-mer CPL scan (100 µM) at 37ºC. After 2 hours, 3x10^4 CD8$^+$ T-cells transduced with the 868 TCR were added and incubated overnight. The supernatant was then harvested and assayed for MIP1β by ELISA.

Figure S1

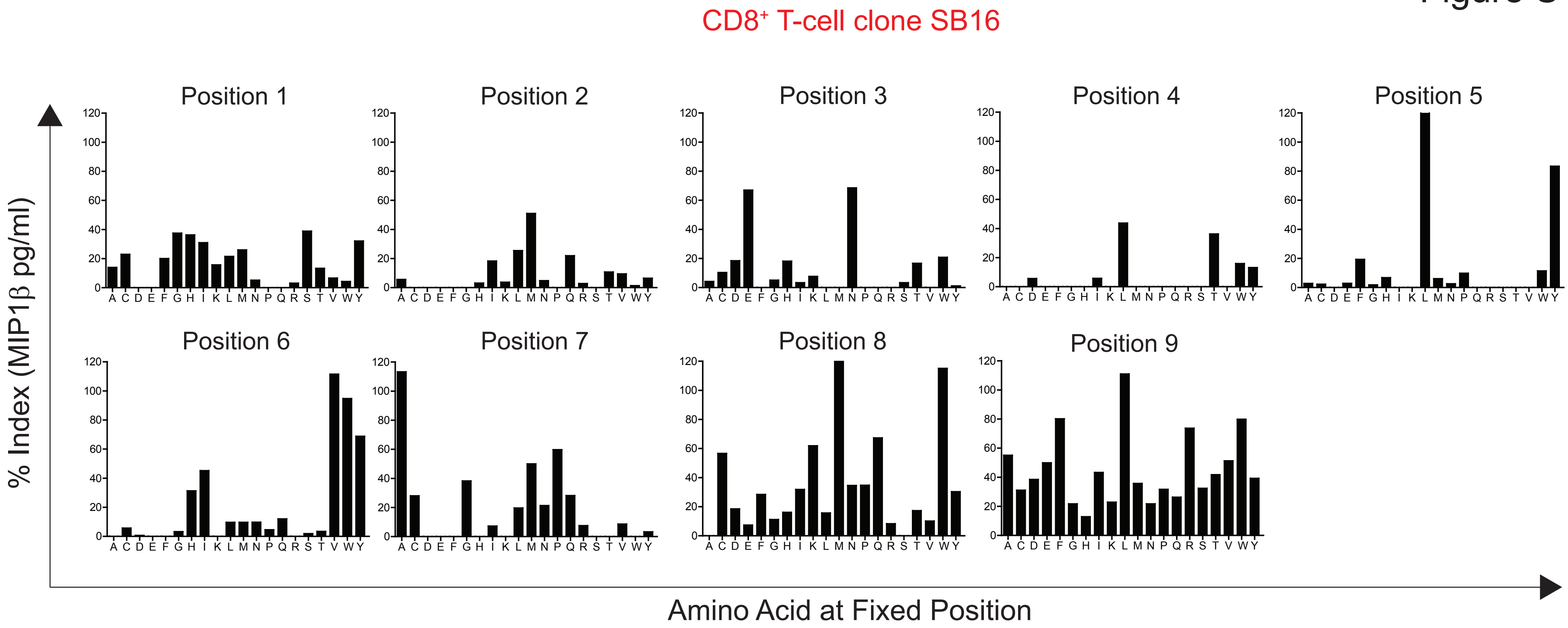
CD8+ T-cell clone SB16
Position 1
Position 2
Position 3
Position 4
Position 5
Position 6
Position 7
Position 8
Position 9
% Index (MIP1β pg/ml)
Amino Acid at Fixed Position
A C D E F G H I K L M N P Q R S T V W Y

Figure S2

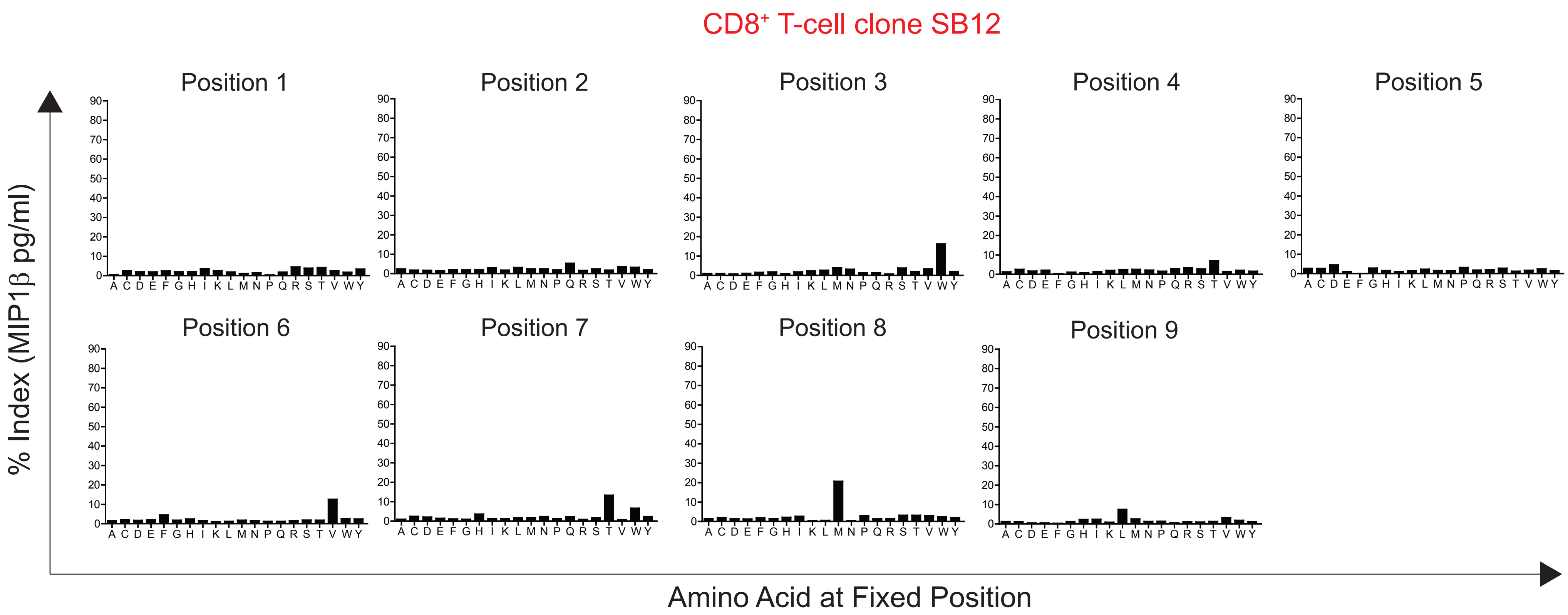
CD8+ T-cell clone SB12
Position 1
Position 2
Position 3
Position 4
Position 5
Position 6
Position 7
Position 8
Position 9
% Index (MIP1β pg/ml)
Amino Acid at Fixed Position
A C D E F G H I K L M N P Q R S T V W Y

Figure S3

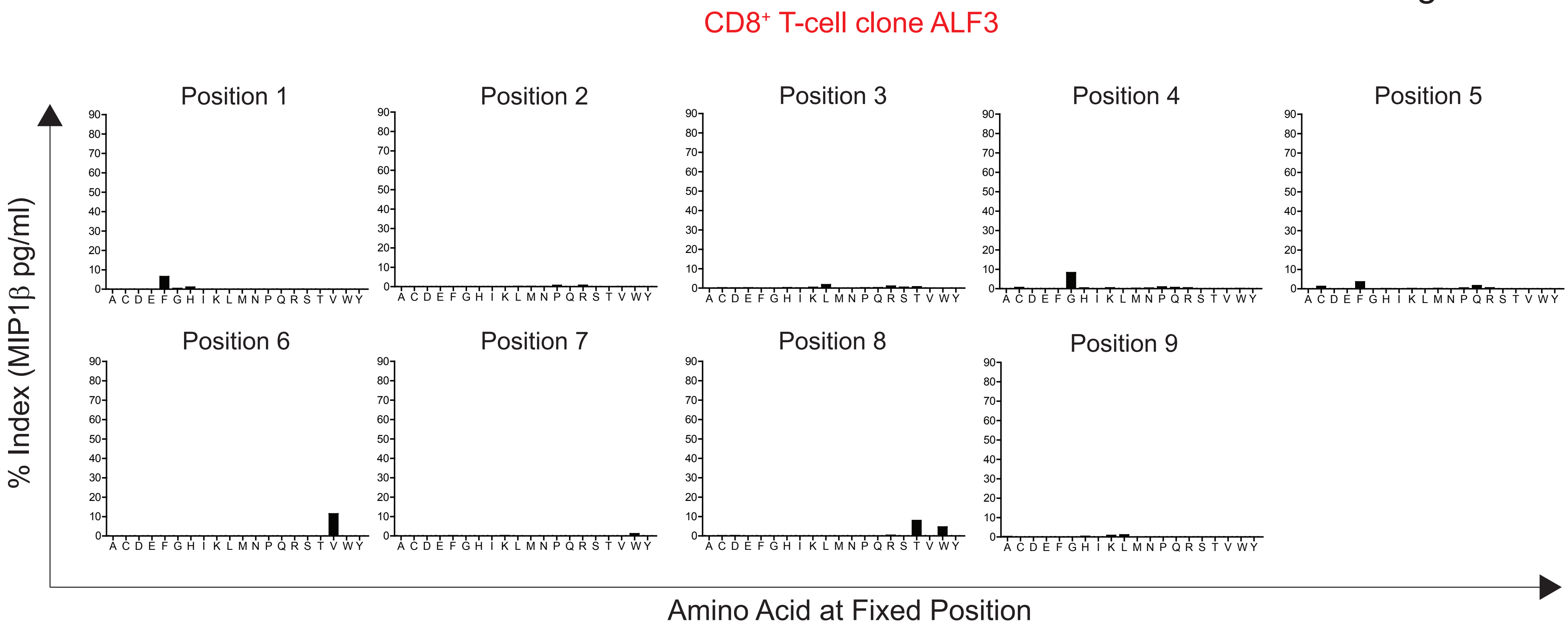
CD8+ T-cell clone ALF3
Position 1
Position 2
Position 3
Position 4
Position 5
Position 6
Position 7
Position 8
Position 9
% Index (MIP1β pg/ml)
Amino Acid at Fixed Position
A C D E F G H I K L M N P Q R S T V W Y

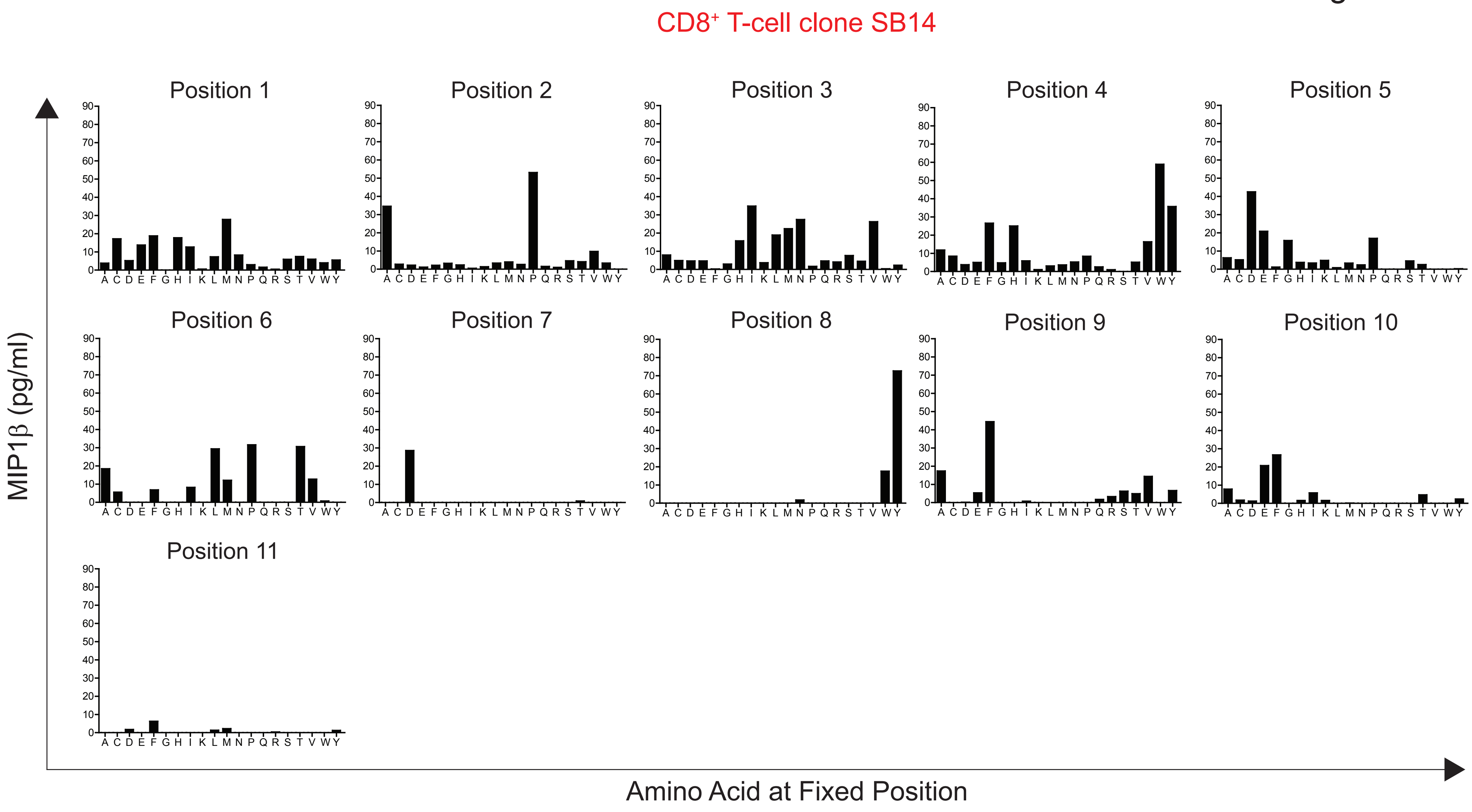
CD8+ T-cell clone SB14
Position 1
Position 2
Position 3
Position 4
Position 5
Position 6
Position 7
Position 8
Position 9
Position 10
Position 11
MIP1β (pg/ml)
Amino Acid at Fixed Position
A C D E F G H I K L M N P Q R S T V W Y
0 10 20 30 40 50 60 70 80 90

Figure S5

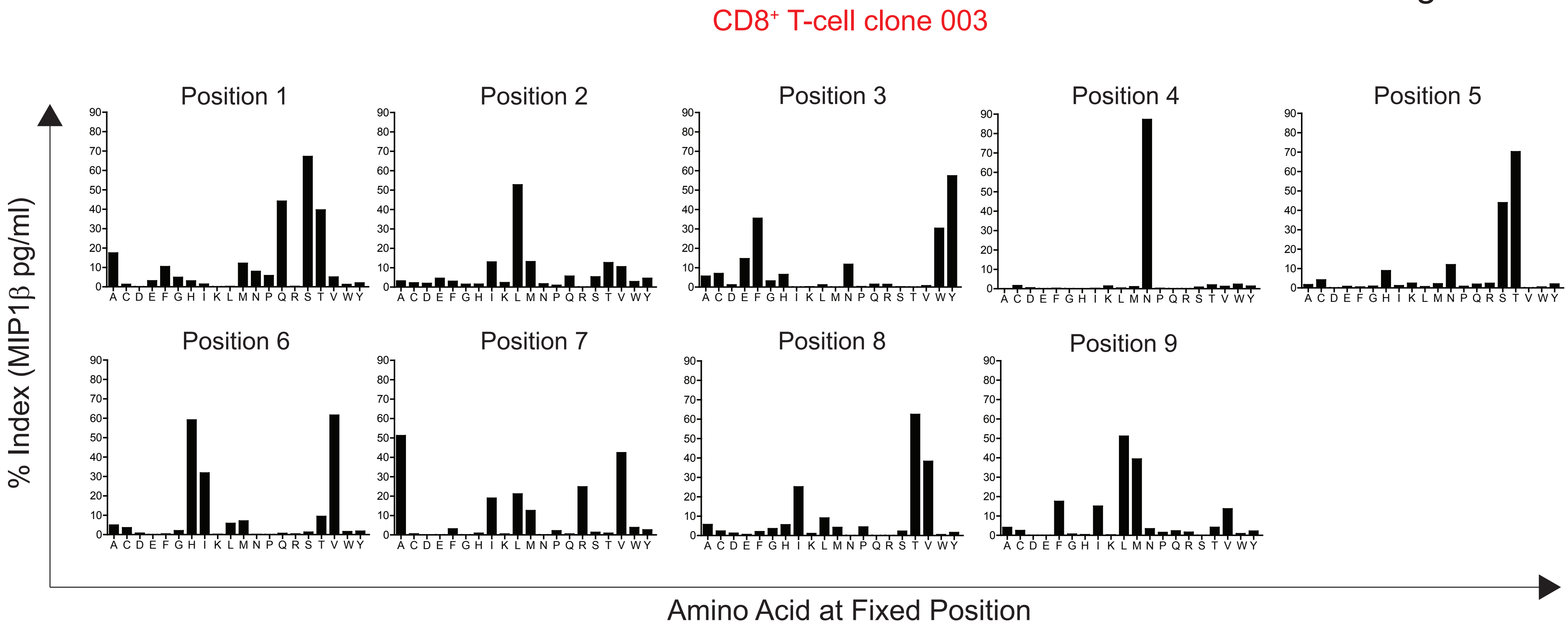
CD8+ T-cell clone 003
Position 1
Position 2
Position 3
Position 4
Position 5
Position 6
Position 7
Position 8
Position 9
% Index (MIP1β pg/ml)
Amino Acid at Fixed Position
A C D E F G H I K L M N P Q R S T V W Y

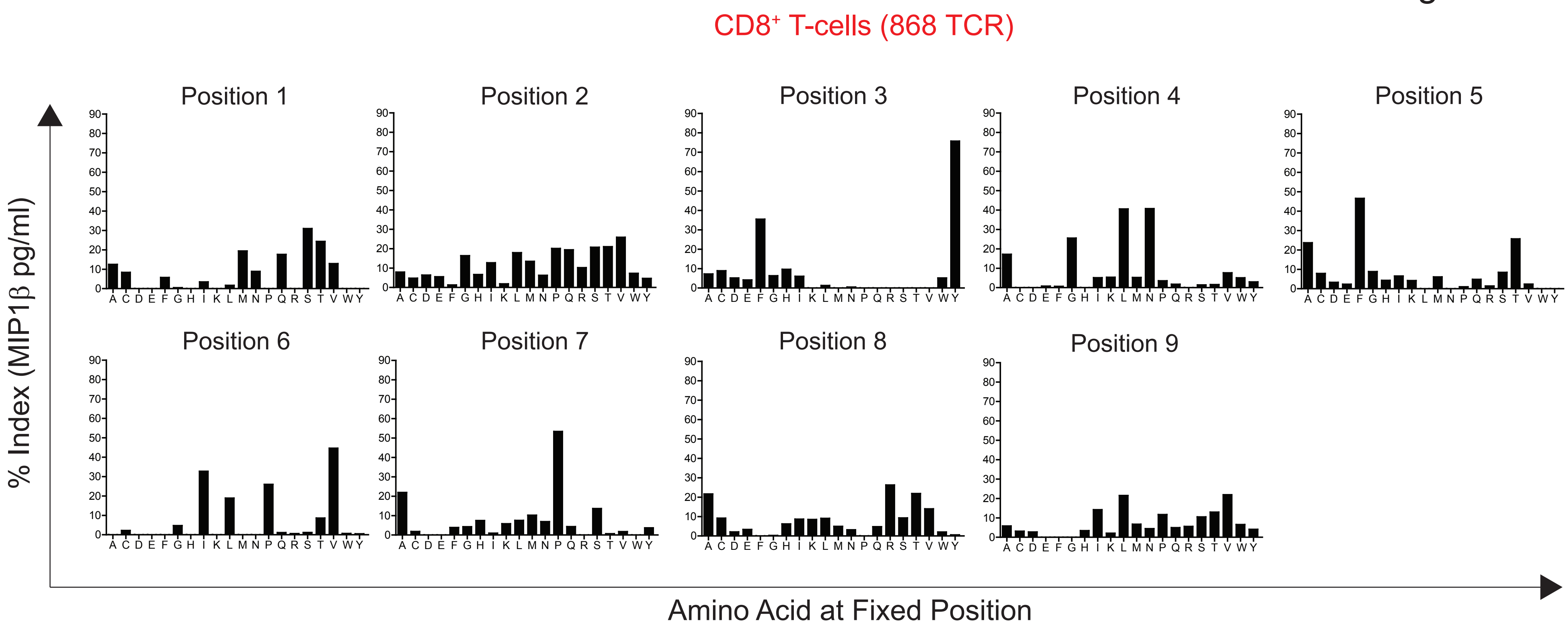
CD8+ T-cells (868 TCR)
Position 1
Position 2
Position 3
Position 4
Position 5
Position 6
Position 7
Position 8
Position 9
% Index (MIP1β pg/ml)
A C D E F G H I K L M N P Q R S T V W Y
Amino Acid at Fixed Position

Supplementary Materials: Supplemental Tables

Rank	Λ	Peptide Sequence	Viral ID
**1**	**-13.21**	**NLVPMVATV**	**HUMAN CYTOMEGALOVIRUS**
2	-14.0927	IVLGLIATA	EASTERN EQUINE ENCEPHALITIS
3	-14.6606	NGVRVLATA	HUMAN METAPNEUMOVIRUS
4	-15.5807	HVLGCLITV	DENGUE VIRUS 2
5	-15.9006	LGMCCIITA	DENGUE VIRUS 2
6	-15.9495	MGVCMIAHV	HUMAN HERPESVIRUS 2
7	-15.9934	MINPLVITT	GUANARITO VIRUS
8	-16.0104	ITNCLLSTA	CRIMEAN-CONGO HEMORRHAGIC FEVER VIRUS
9	-16.1097	KDLVLLATI	HUMAN ADENOVIRUS B/D/E
10	-16.2304	NIVCPLCTL	HUMAN PAPILLOMAVIRUS 1A
11	-16.2601	MILGPISTA	MIDDELBURG VIRUS
12	-16.3254	IIVGCVPTI	HUMAN PAPILLOMAVIRUS 179
13	-16.52006	RLTGLLATS	HUMAN HERPESVIRUS 1
14	-16.5221	ILLACLATL	ALKHUMRA HEMORRHAGIC FEVER VIRUS
15	-16.5859	VVNPVVATA	HUMAN CYTOMEGLAOVIRUS
16	-16.5929	CMLRLVCTA	MOLLUSCUM CONTAGIOSUM VIRUS
17	-16.7047	KTNPLPATP	HUMAN HERPESVIRUS 1
18	-16.735	IVLRMVIYV	HUMAN IMMUNODEFICIENCY VIRUS 2
19	-16.779	MMLVPLITV	MONKEYPOX VIRUS
20	-16.8048	FILGIIITV	VARIOLA VIRUS

Table S1A: CPL-driven search of the human viral database for E7NLV (“Index” peptide sequence in boldface).

Rank	Λ	Peptide Sequence	Viral ID
**1**	**-15.3323**	**GLCTLVAML**	**HUMAN HERPESVIRUS 4**
2	-15.803	KMNTLVAQS	HUMAN TMEV-LIKE CARDIOVIRUS
3	-16.2656	SLNTLQPML	HUMAN PARAINFLUENZAVIRUS 1/3
4	-16.7366	LLEYLYMMR	TANAPOX VIRUS
5	-16.7367	GQNLLYANS	HUMAN ADENOVIRUS B/C
6	-16.7949	SLNLPVAKL	HUMAN IMMUNODEFICIENCY VIRUS 2
7	-16.8288	ILNTLVAYQ	HUMAN HERPESVIRUS 7
8	-16.9413	SLGLLVAWA	CERCOPITHECINE HERPESVIRUS 1
9	-17.0839	LLDTLVMQL	HUMAN HERPESVIRUS 8
10	-17.1431	LLELYVPKS	HUMAN HERPESVIRUS 7
11	-17.2793	TINTLIAMK	TANAPOX VIRUS
12	-17.3584	LYNLLVLWL	HUMAN HERPESVIRUS 6A
13	-17.3613	TLDTLVAMK	MOLLUSCUM CONTAGIOSUM VIRUS
14	-17.4863	ILWLLVMIF	HUMAN CORONAVIRUS Nl63
15	-17.5593	LQELLIQQW	SARS CORONAVIRUS
16	-17.5627	ILNLLVIQR	ISFAHAN VIRUS, CHANDIPURA VIRUS, VESICULAR STOMATITIS INDIANA VIRUS
17	-17.5649	NAELLVAME	INFLUENZA A VIRUS
18	-17.5647	SLNLPVAKV	HUMAN IMMUNODEFICIENCY VIRUS 1
19	-17.5699	TMDTLIAMK	MONKEYPOX VIRUS
20	-17.6729	TQELLYAYT	DHORI VIRUS

Table S1B: CPL-driven search of the human viral database for SB16 (“Index” peptide sequence in boldface).

Rank	Λ	Peptide Sequence	Viral ID
1	-17.2068	HPVAEADYFEY	HUMAN HERPESVIRUS 4
2	-17.3948	HPVGDADYFEY	HUMAN HERPESVIRUS 4
**3**	**-18.1085**	**HPVGEADYFEY**	**HUMAN HERPESVIRUS 4**
4	-20.203	SPQWAADYAFL	CERCOPITHECINE HERPESVIRUS 1
5	-20.3323	SPRWAADYAFL	CERCOPITHECINE HERPESVIRUS 16
6	-21.2014	FVNFNVDWVFF	HUMAN CORONAVIRUS NL63
7	-22.0733	LASLGVDYSEF	SUID HERPESVIRUS 1
8	-22.5825	TPNYDIDLAFF	HUMAN ADENOVIRUS B/E
9	-22.6401	YPNWDTIYYED	HUMAN PAPILLOMAVIRUS 167
10	-22.7321	LHAVPIDYFFL	ADULT DIARRHEAL ROTAVIRUS
11	-23.0102	PCMVGPDYAYF	MIDDLE EAST RESPIRATORY SYNDROME CORONAVIRUS
12	-23.0682	KNVWDVDYSAF	FOOT-AND-MOUTH DISEASE VIRUS
13	-23.1986	RNVWDVDYSAF	FOOT-AND-MOUTH DISEASE VIRUS
14	-23.3088	TGCCSTDYFEM	HUMAN CORONAVIRUS 229E
15	-23.5894	EVLREADYSED	ASTROVIRUS VA1
16	-23.5944	CLLLSTDWVEF	SAIMIRIINE HERPESVIRUS 2
17	-23.6211	DAIVEADYSAN	TIOMAN VIRUS
18	-23.7977	SVAPEVDWVAF	HUMAN CORONAVIRUS NL63
19	-23.9037	DAVVEADYSAN	MENANGLE VIRUS
20	-23.9122	PTSVPLDWAAF	HUMAN HERPESVIRUS 2

Table S1C: CPL-driven search of the human viral database for SB14 (“Index” peptide sequence in boldface).

Rank	Λ	Peptide Sequence	Viral ID
1	-34.9645	NVASLUGSTVREY	MOLLUSCUM CONTAGIOSUM VIRUS
2	-35.3404	LIENVASLUGSTV	MOLLUSCUM CONTAGIOSUM VIRUS
3	-35.6833	VASLUGSTVREYT	MOLLUSCUM CONTAGIOSUM VIRUS
4	-35.8699	SLUGSTVREYTQM	MOLLUSCUM CONTAGIOSUM VIRUS
5	-35.9034	ASLUGSTVREYTQ	MOLLUSCUM CONTAGIOSUM VIRUS
6	-36.0576	LLIENVASLUGST	MOLLUSCUM CONTAGIOSUM VIRUS
7	-36.481	VLLIENVASLUGS	MOLLUSCUM CONTAGIOSUM VIRUS
8	-36.6355	YLAPAPQTPLAFY	HUMAN HERPESVIRUS 2
9	-36.6396	APLPCFQNNCLFL	ENCEPHALOMYOCARDITIS VIRUS
10	-36.6518	SAAPAFQAPRFGL	WHATAROA VIRUS
11	-36.6783	HPFGSPQTDNPCY	TORQUE TENO VIRUS 19
**12**	**-36.7192**	**LPEPLPQGQLTAY**	**HUMAN HERPESVIRUS 4**
13	-36.7205	SAVKSPQAPLVLC	JUNIN ARENAVIRUS
14	-36.7216	LALPAPPSQPFPW	HUMAN T-LYMPHOTROPIC VIRUS 2
15	-36.7294	SAIKSPQAPLVLC	JUNIN VIRUS
16	-36.7331	WPEPTFPSRWYWL	HUMAN HERPESVIRUS 6A
17	-36.7375	WTLGLFQVSHGIF	HUMAN HERPESVIRUS 8
18	-36.7405	LLSPLPMTPEPTL	HUMAN HERPESVIRUS 6B
19	-36.7474	TVQGPFSAACGLF	HUMAN ADENOVIRUS A/F
20	-36.7475	TPMPPPQGPPTAM	HUMAN HERPESVIRUS 4

Table S1D: CPL-driven search of the human viral database for SB27 (“Index” peptide sequence in boldface).

Rank	Λ	Peptide Sequence	Viral ID
**1**	**-8.8549**	**SLYNTVATL**	**HUMAN IMMUNODEFICIENCY VIRUS 1**
2	-9.3363	SLFNTVATL	HUMAN IMMUNODEFICIENCY VIRUS 1
3	-9.3448	SLYNTVAVL	HUMAN IMMUNODEFICIENCY VIRUS 1
4	-9.8261	SLFNTVAVL	HUMAN IMMUNODEFICIENCY VIRUS 1
5	-10.006	SLYNTIAVL	HUMAN IMMUNODEFICIENCY VIRUS 1
6	-10.676	SLFNTIVVL	HUMAN IMMUNODEFICIENCY VIRUS 1
7	-11.038	SLHNTVATL	HUMAN IMMUNODEFICIENCY VIRUS 1
8	-12.608	SLYNAVATL	HUMAN IMMUNODEFICIENCY VIRUS 1
9	-13.286	SLYNAVVVL	HUMAN IMMUNODEFICIENCY VIRUS 1
10	-13.452	SLFNTTAIV	HUMAN IMMUNODEFICIENCY VIRUS 1
11	-14.216	SIDNTVATL	HUMAN PAPILLOMAVIRUS
12	-14.397	SLWNAIAVL	HUMAN IMMUNODEFICIENCY VIRUS 1
13	-14.531	TIFNTLLTL	ROTAVIRUS A
14	-14.586	SLWNAIVVL	HUMAN IMMUNODEFICIENCY VIRUS 1
15	-14.599	SMFNKVAVL	ROTAVIRUS A
16	-14.633	SLFNLVAVL	HUMAN IMMUNODEFICIENCY VIRUS 1
17	-14.669	SLGNTHVAM	HUMAN IMMUNODEFICIENCY VIRUS 1
18	-15.021	SMFNKVAIL	ROTAVIRUS A
19	-15.249	SLYNTVCVI	HUMAN IMMUNODEFICIENCY VIRUS 2
20	-15.335	QVYNTAIIL	YABA-LIKE DISEASE VIRUS

Table S1E: CPL-driven search of the human viral database for 003 (“Index” peptide sequence in boldface).

Rank	Λ	Peptide Sequence	Viral ID
**1**	**-15.1273**	**SLYNTVATL**	**HUMAN IMMUNODEFICIENCY VIRUS 1**
2	-15.2083	SLYNAVATL	HUMAN IMMUNODEFICIENCY VIRUS 1
3	-15.5765	SLYNTVAVL	HUMAN IMMUNODEFICIENCY VIRUS 1
4	-15.8067	SSYGAPPAP	SAPOVIRUS
5	-15.8858	SLYNTIAVL	HUMAN IMMUNODEFICIENCY VIRUS 1
6	-15.8864	SLFNTVATL	HUMAN IMMUNODEFICIENCY VIRUS 1
7	-16.3355	SLFNTVAVL	HUMAN IMMUNODEFICIENCY VIRUS 1
8	-16.4996	VLYNFVSTP	SAIMIRIINE HERPESVIRUS 2
9	-16.5179	SLYNALAVL	HUMAN IMMUNODEFICIENCY VIRUS 1
10	-16.7137	ASYGAPPAP	SAPOVIRUS
11	-16.9083	TTFAAVAAV	COXSACKIEVIRUS A24
12	-16.957	SVYNFLSKT	MONKEYPOX VIRUS, VACCINIA VIRUS, VARIOLA VIRUS
13	-17.0372	SIFNIVPRT	MONKEYPOX VIRUS, VACCINIA VIRUS, VARIOLA VIRUS
14	-17.1894	SLHNTVATL	HUMAN IMMUNODEFICIENCY VIRUS 1
15	-17.2614	TGYYAVPTV	HUMAN HERPESVIRUS 6A
16	-17.3227	SNYLQPPRL	HUMAN HERPESVIRUS 3
17	-17.4171	AMYNSVPLV	CRIMEAN-CONGO HEMORRHAGIC FEVER VIRUS
18	-17.4273	TGYNFPHKL	HUMAN PAPILLOMAVIRUS
19	-17.4426	TTYLALMAT	DENGUE VIRUS 1
20	-17.496	TPYNFIAKN	VACCINIA VIRUS

Table S1F: CPL-driven search of the human viral database for 868 (“Index” peptide sequence in bold face).

Rank	Λ	Peptide Sequence	Human Self Protein ID
1	-13.8709	LSGKWLMHL	UPF0696 protein C11orf68
2	-14.6509	LTQKWCHTL	F-box/LRR-repeat protein 6
3	-14.7419	LLGKFCTTF	cubilin precursor
4	-14.9425	IILKFLARI	semaphorin-6A
5	-14.9697	LLGLFLFQL	semaphorin-4A
6	-15.006	MTGKFCIIL	olfactory receptor 11H6
7	-15.1703	IIGKFCTAL	phospholipase A1 member A
**8**	**-15.2091**	**ILAKFLHWL**	**telomerase reverse transcriptase**
9	-15.3153	LTGAFLFSL	ATP-sensitive inward rectifier potassium channel 10/15
10	-15.4063	LQGLFLFSL	dynein heavy chain 3, axonemal
11	-15.4983	NIGKFLNRI	protein strawberry notch homolog
12	-15.5045	IIGKFQFTV	protein-glutamine gamma-glutamyltransferase K
13	-15.5077	ITRKHLWRL	coiled-coil domain-containing protein 108
14	-15.5221	MVGKFGVTA	Solute carrier family 22
15	-15.54	MSGWFLRRT	Putative DNA repair and recombination protein RAD26
16	-15.5526	ILGKHGFFV	phosphoglucomutase-1
17	-15.5801	FLGKSLFSL	epidermal retinol dehydrogenase 2
18	-15.6492	MLGLFLYSL	otoferlin
19	-15.6538	MTGLWIFTI	N-formyl peptide receptor 3
20	-15.7099	LIQKHLVRL	E3 ubiquitin-protein ligase UBR1

Table S2A: CPL-driven search of the human self database for ILA1 (“Index” peptide sequence in boldface).

Rank	Λ	Peptide Sequence	Human Self Protein ID
1	-16.8944	LLAGIGTVPI	solute carrier organic anion transporter
2	-17.418	ILEGIGILAV	anoctamin-3
3	-17.5337	LLLGIGILVL	bone marrow stromal antigen 2
4	-17.6102	FLAGLGLLVI	translocon-associated protein subunit alpha
5	-17.6562	AAAAIFVIII	MHC class I polypeptide-related sequence A
6	-17.7312	FVAGIFLLVV	protocadherin Fat 1 precursor
7	-17.9409	FITGKGIVAI	leiomodin-3
8	-18.0041	LITGLGIISV	adenosine receptor A3
9	-18.013	ILLGIGIYAL	transmembrane and coiled-coil domain-containing protein 2
10	-18.0426	LLAGLGILAG	provirus ancestral Env polyprotein preproprotein
11	-18.1265	ISAAIWIVVG	putative P2Y purinoceptor 10
12	-18.1456	IAAGTGIVIL	transmembrane 7 superfamily member 4
13	-18.228	FITATGVVKL	serine/threonine-protein kinase Nek6
14	-18.2536	ITAGLPVKVV	amyloid protein-binding protein 2
15	-18.2816	LKTGIGVIRM	neuropilin and tolloid-like protein 2 precursor
16	-18.3184	SLTGLGVVKV	E3 ubiquitin-protein ligase HERC2
17	-18.3348	WTAPIGVISL	uncharacterized protein C5orf4
18	-18.3916	FITGTGILAL	tropomodulin-2
19	-18.399	AGTGIGLMVL	intermediate conductance calcium-activated potassium channel protein 4
20	-18.4265	ILEGIGILSV	anoctamin-4

Table S2B: CPL-driven search of the human self database for MEL5 (EAAGIGILTV ranked 55).
